# Biocontrol Capabilities of *Bacillus subtilis* E11 against *Aspergillus flavus In Vitro* and for Dried Red Chili (*Capsicum annuum* L.)

**DOI:** 10.3390/toxins15050308

**Published:** 2023-04-26

**Authors:** Shenglan Yuan, Yongjun Wu, Jing Jin, Shuoqiu Tong, Lincheng Zhang, Yafei Cai

**Affiliations:** Key Laboratory of Plant Resource Conservation and Germplasm Innovation in Mountainous Region (Ministry of Education), College of Life Sciences/Institute of Agro-Bioengineering, Guizhou University, Guiyang 550025, China

**Keywords:** AFB_1_, *Bacillus subtilis* E11, *Aspergillus flavus*, detoxification, dried red chili

## Abstract

As a condiment with extensive nutritional value, chili is easy to be contaminated by *Aspergillus flavus* (*A. flavus*) during field, transportation, and storage. This study aimed to solve the contamination of dried red chili caused by *A. flavus* by inhibiting the growth of *A. flavus* and detoxifying aflatoxin B_1_ (AFB_1_). In this study, *Bacillus subtilis* E11 (*B. subtilis*) screened from 63 candidate antagonistic bacteria exhibited the strongest antifungal ability, which could not only inhibit 64.27% of *A. flavus* but could also remove 81.34% of AFB_1_ at 24 h. Notably, scanning electron microscopy (SEM) showed that *B. subtilis* E11 cells could resist a higher concentration of AFB_1_, and the fermentation supernatant of *B. subtilis* E11 could deform the mycelia of *A. flavus*. After 10 days of coculture with *B. subtilis* E11 on dried red chili inoculated with *A. flavus*, the mycelia of *A. flavus* were almost completely inhibited, and the yield of AFB_1_ was significantly reduced. Our study first concentrated on the use of *B. subtilis* as a biocontrol agent for dried red chili, which could not only enrich the resources of microbial strains for controlling *A. flavus* but also could provide theoretical guidance to prolong the shelf life of dried red chili.

## 1. Introduction

China has the largest production of chili worldwide [1], in which dried red chili (*Capsicum annuum* L.) is one of the common condiments. However, it is vulnerable to pollution by aflatoxins (AFTs) as other crops [2]. The contamination and growth of *Aspergillus flavus* (*A. flavus*) may occur anywhere and anytime during the pre-harvest or post-harvest of chili [3]. After contamination with *A. flavus*, chili may not only cause serious economic losses but may also endanger human health. For instance, Rajendran et al. measured the AFTs in 42 samples of chili grown and marketed in Tamil Nadu. The results showed that approximately 66.7% of the samples collected from retail stores exceeded the European Commission standard limit (10 μg/kg). The content of AFTs in the samples collected across the value chain ranged from 3.83 to 37.80 μg/kg [4,5]. Therefore, it is urgent to globally solve the challenges of *A. flavus* pollution and the production of AFTs.

*A. flavus* is a common saprophytic fungus that widely exists in nature. AFTs are secondary toxic metabolites that are mainly produced by *A. flavus* and *Aspergillus parasiticus* [6]. To date, more than 18 types of AFTs have been found [7], mainly including aflatoxin B_1_ (AFB_1_), aflatoxin B_2_ (AFB_2_), aflatoxin G_1_ (AFG_1_), and aflatoxin G_2_ (AFG_2_) [8,9]. AFB_1_ has been classified as a group I carcinogen by the International Agency for Research on Cancer (IARC) because of its strong toxicity [10]. The toxicity of AFB_1_ has been demonstrated to act on most animals [11], which is carcinogenic, teratogenic, and mutagenic [12]. AFB_1_ can also cause irreversible effects on the kidneys, pancreas, bladder, bones, viscera, and central nervous system. Among them, damage to the liver is the most well-known concern. Besides, it may cause a series of chronic and acute diseases [13]. For crops, AFB_1_ pollution exists widely, which usually occurs during pre and post-harvesting of regional crops and food commodities [14].

The AFTs synthesis gene cluster is located on chromosome 3, with a DNA region of about 70 kb [15]. Based on the current research results, the biosynthesis of AFTs is regulated by at least 53 genes, containing 39 positive regulatory genes, 12 negative regulatory genes, and two other regulatory genes [16]. It is noteworthy that *aflR* and *aflS* are two regulatory genes in the biochemical synthesis pathway of AFB_1_. The *aflR* gene is a transcriptional activator of the AFTs gene cluster, which is necessary to activate the expression of other AFTs genes. The *aflR* gene is responsible for encoding the zinc-finger transcription factor. The *aflS* gene is located in the attachment of the *aflR* gene and is a transcriptional co-activator of the AFTs gene cluster. The ketoreductase encoded by the *aflD* gene could convert norsolorinic acid (NOR) into averantin (AVN), belonging to structural genes [17,18,19]. These three genes play a crucial role in the synthesis of AFTs.

At present, the control strategies of AFB_1_ mainly include physical (adsorption, irradiation, thermal treatment, and mechanical sorting), chemical (curcumin, salts, acids, and alkaline), and biological methods [20,21,22,23,24]. The control of AFB_1_ based on physical and chemical methods cannot be widely used because of the nutrient loss, chemical residue, sensory quality reduction, and high cost. In contrast, biodegradation is more moderate and environmentally safe, with less impact on food quality [25,26]. Thus, biological methods to inhibit the growth of *A. flavus* and the production of AFTs have noticeably attracted scholars’ attention. It was reported that some species of bacteria, fungi, and algae could well prevent the growth of *A. flavus* and the production of toxins [27]. The reported biological control methods of AFB_1_ have mainly used bacteria or fungi and antagonistic substances produced by their metabolism to adsorb AFB_1_, degrade it enzymatically or convert AFB_1_ into other substances with lower toxicity [28]. For instance, Cai et al. [29] screened a *Stenotrophomonas acidoaminiphila* strain that could reduce AFB_1_ concentration, and they demonstrated that this strain could degrade AFB_1_ through the combination of enzymes and micro-molecular oxides. A recent study showed that the supernatant of *Trichoderma reesei* could effectively degrade AFB_1_ with a concentration of 500 ng/kg and with a degradation rate of 91.8%, while the degradation rates of intracellular components and mycelial adsorption were 19.5% and 8.9%, respectively. It was also proved that the degradation products of this strain were nontoxic [30]. In another study, Zhao et al. [31] purified an extracellular enzyme called myxobacteria aflatoxin degradation enzyme (MADE) from *Myxococcus fulvus* ANSM068, which could simultaneously degrade AFB_1_ (71.89%), AFG_1_ (68.13%), and aflatoxin M_1_ (AFM_1_) (63.82%).

*Bacillus subtilis* (*B. subtilis*) and Lactic acid bacteria (LAB) are commonly known as generally recognized as safe (GRAS) [32,33], which are recognized as biological control agents against a variety of fungi [34,35]. Ren et al. [36] demonstrated that *Bacillus* was found in 21% of the research articles on controlling aflatoxigenic fungi and AFB_1_. Because of its fast growth rate, and its ability to produce a wide range of antifungal compounds, *Bacillus* has been widely used in the control of aflatoxigenic fungi and the production of AFTs. For instance, Zhao et al. [37] screened a strain from 22 strains of *Lactobacillus plantarum* that could completely inhibit the growth of *A. flavus* at a concentration of 5 × 10^5^ CFU/mL and reduced the yield of AFTs by 93%. The cell-free supernatant of another *Lactobacillus plantarum* UM55 inhibited the production of AFTs by 91% [38]. According to Suresh et al.’s study [28], *B. subtilis* could degrade 60% of AFB_1_. Another study showed that the treatment of pistachios with *B. subtilis* UTBSP1 could reduce the growth of *A. flavus* R5 and the production of AFB_1_ on pistachios [39].

In conclusion, although biological control is one of the most promising methods to solve the pollution of *A. flavus* and AFB_1_, it may be inapplicable to some types of food. Some scholars have shown that the biological detoxication of mycotoxins and their ability to alter the chemical structures deserve further attention [40]. On the other hand, as antagonistic bacteria that inhibit the growth of *A. flavus* and detoxify AFB_1_, they could not promptly and effectively inhibit the growth of *A. flavus*, which would ultimately lead to the accumulation of AFB_1_ [41]. At present, the control of the production of AFTs mainly depends on two key points, one is to prevent the growth of AFTs-producing fungi, and the other is to remove toxic compounds through various methods to achieve the purpose of detoxification in case of pollution [42]. Therefore, the present study aimed to screen bacteria with strong antifungal activity and detoxification capability of AFB_1_ in order to provide a new strategy to solve the pollution of *A. flavus* and AFB_1_ on dried red chili.

In the present study, we screened a strain of *B. subtilis* E11 that exhibited significant biological control capabilities against *A. flavus* in vitro and on dried red chili (Denglongjiao, one of the main varieties of chili cultured in the Guizhou province of China). Therefore, the results of the present study may provide an effective theoretical basis for the development of biological preservatives that can effectively control the growth and toxicity of *A. flavus* and broaden the biological control pathway of *A. flavus* and AFB_1_ in dried red chili storage.

## 2. Results

### 2.1. Screening of Strains with Biological Antagonistic Ability to A. flavus

**Figure 1:**
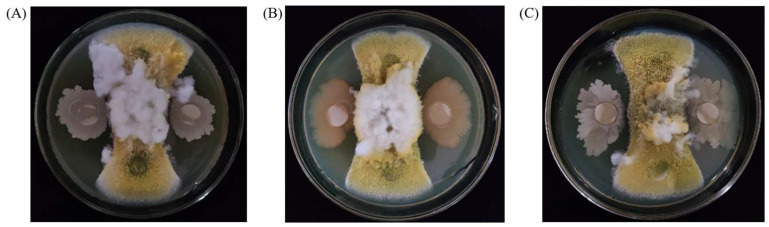
Inhibitory effects of *Bacillus subtilis* E11 (*B. subtilis*), *B. subtilis* V1J1, and *B. subtilis* 9932 on *Aspergillus flavus* (*A. flavus*). (**A**–**C**) represented the inhibition rates of *B. subtilis* E11, *B. subtilis* V1J1, and *B. subtilis* 9932 on *A. flavus* mycelia, respectively. Each Potato Dextrose Agar (PDA) plate had five filter paper discs. The central filter paper disc was inoculated with *A. flavus* spore suspension, the two vertical filter paper discs were inoculated with water as the control group, and the two horizontal filter paper discs were inoculated with a bacterial solution as the experimental group.

Preliminary screening and rescreening results of antagonistic bacteria are shown in Table S1 and Figure S1. Among 63 strains of LAB and *B. subtilis*, three strains of *B. subtilis*, including E11, V1J1, and 9932, were finally selected as antagonistic bacteria for the next study according to their inhibitory abilities to *A. flavus* (Figure 1). According to the results of confronting incubation, the inhibitory rates of *B. subtilis* E11, V1J1, and 99,332 on the growth of *A. flavus* mycelia were 64.27 ± 2.06%, 62.57 ± 1.85%, and 69.31 ± 5.45%, respectively. Considering that *B. subtilis* E11, V1J1, and 9932 had strong inhibitory effects on the mycelia of *A. flavus*, we believe that these three strains have potential in the biological control of *A. flavus* and should be further studied.

### 2.2. The Abilities of B. subtilis E11, B. subtilis V1J1, and B. subtilis 9932 to Remove AFB_1_

**Figure 2:**
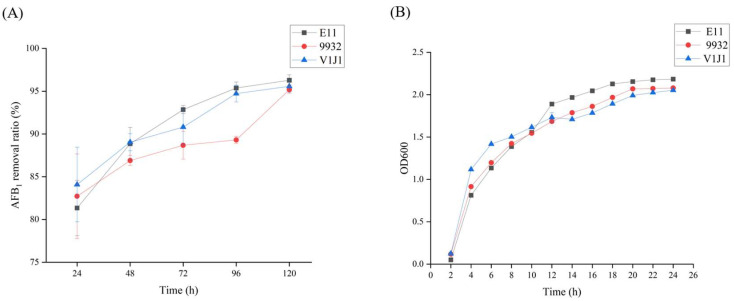
Determination of aflatoxin B_1_ (AFB_1_) removal ability and growth curve of *B. subtilis* E11, *B. subtilis* V1J1 and *B. subtilis* 9932. (**A**) The abilities of *B. subtilis* E11, *B. subtilis* V1J1, and *B. subtilis* 9932 to degrade AFB_1_ at different time points. (**B**) The growth curves of *B. subtilis* E11, *B. subtilis* V1J1, and *B. subtilis* 9932 at different time points. Bars were expressed as means ± standard deviation (SD) from three biological repeats.

The abilities of *B. subtilis* E11, *B. subtilis* 9932, and *B. subtilis* V1J1 to remove AFB_1_ were further detected by an ELISA kit at different time points. It can be seen from Figure 2A and Table S2 that the ability of *B. subtilis* E11 to remove AFB_1_ was slightly higher than that of *B. subtilis* 9932 and *B. subtilis* V1J1. Afterward, according to the growth curves of the three strains, it was found that the number of cells in the platform stage of *B. subtilis* E11 was higher than that in the other two strains, which could lead to more secondary accumulated metabolites (Figure 2B). Therefore, *B. subtilis* E11 was selected for further study.

### 2.3. The Biodegradation Ability of AFB_1_ by Culture Supernatant, Cells, and Cell Lysates

**Figure 3:**
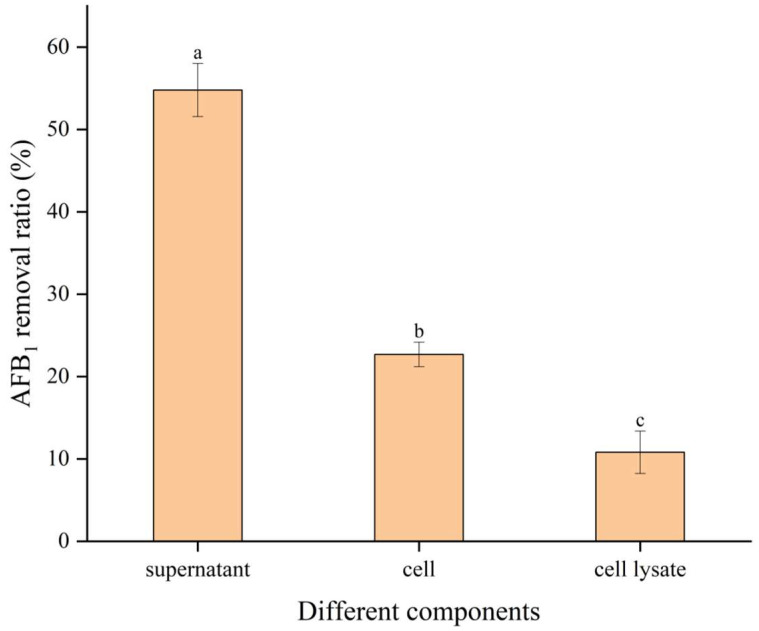
The abilities of different components of *B. subtilis* E11 to degrade AFB_1_. Values were expressed as means ± SD; a, b, and c indicated significant differences among different components (*p* < 0.05).

AFB_1_ was added to the fermentation supernatant, cells, and cell lysates of *B. subtilis* E11 and cultured for 24 h, in which the results showed that the degradation rate of AFB_1_ in the culture supernatant of *B. subtilis* E11 was the highest (54.78%) (Figure 3). Moreover, degradation rates of 22.68% and 10.81% were obtained when AFB_1_ was treated with cells and cell lysates, respectively. It was revealed that the fermentation supernatant of *B. subtilis* E11 had the greatest contribution to the degradation of AFB_1_.

### 2.4. The Effects of B. subtilis E11 on aflR, aflS, and aflD Key Genes for AFB_1_ Production

**Figure 4:**
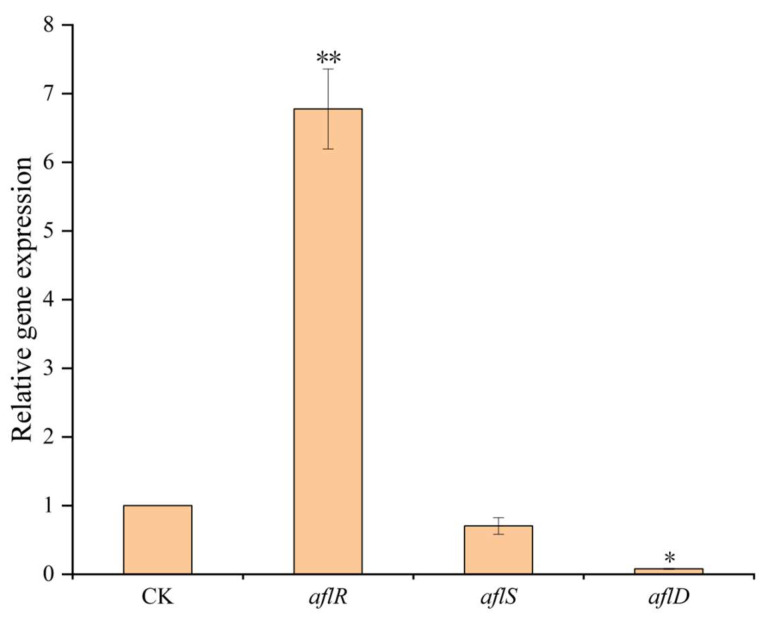
Relative expression levels of *aflR*, *aflS*, and *aflD* genes. CK indicated the control group. Values were expressed as means ± SD. Significantly differential gene expression was indicated as * *p*-value < 0.05, ** *p*-value < 0.01.

Based on the results of the expression of three key genes in the AFB_1_ synthesis pathway, the fermentation supernatant of *B. subtilis* E11 could significantly increase the *aflR* expression level and significantly repress the *aflD* expression level, while the *aflS* expression level slightly decreased compared with the control group (Figure 4).

### 2.5. The Ultrastructural Changes of B. subtilis E11 and A. flavus

**Figure 5:**
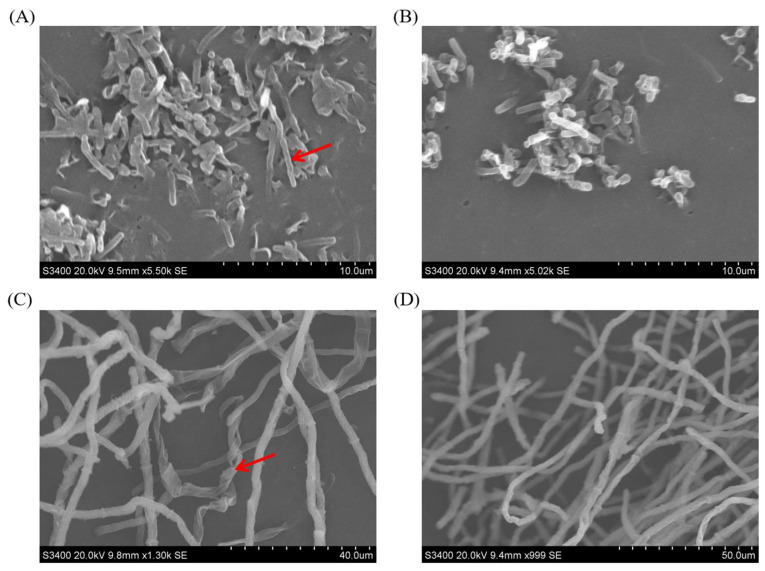
Ultrastructural changes of *B. subtilis* E11 and *A. flavus*. (**A**) Morphology of *B. subtilis* treated with AFB_1_; (**B**) Morphology of untreated *B. subtilis* E11 was used as a control group; (**C**) Mycelia morphology of *A. flavus* cocultured with fermentation supernatants of *B. subtilis* E11; (**D**) Mycelia morphology of untreated *A. flavus* was used as a control group. The red arrows in Figure 5A,C represented the microstructural changes of *B. subtilis* E11 and *A. flavus*, respectively.

The ultrastructural changes of *B. subtilis* E11 treated with AFB_1_ and *A. flavus* treated with *B. subtilis* E11 fermentation supernatant were observed by scanning electron microscopy (SEM) (Figure 5). Figure S2 shows the effect of the fermentation supernatant of *B. subtilis* E11 on *A. flavus* mycelia. It was revealed that *A. flavus* in the control group could form a mycelia ball after 48 h of culture, while the mycelia of *A. flavus* treated with the supernatant of *B. subtilis* E11 presented a loose flocculent structure and could not form mycelia ball. The purpose of using SEM to analyze the ultrastructural changes of *B. subtilis* E11 was to indicate whether cells of *B. subtilis* E11 could resist a high concentration of AFB_1_. The ultrastructural changes of *B. subtilis* E11 after coculture with AFB_1_ showed that the *B. subtilis* cells of E11 became irregular, and the surface became rough with slight wrinkles after 24 h (Figure 5A). The cells of *B. subtilis* E11 without AFB_1_ treatment were regularly rod-shaped, and the cell surface was smooth (Figure 5B). The mycelia morphology of *A. flavus* cocultured with fermentation supernatants of *B. subtilis* E11 had obvious folds and deformation (Figure 5C). However, the mycelia of *A. flavus* in the control group had a smooth appearance without wrinkles (Figure 5D). Therefore, *B. subtilis* E11 cells not only have the ability to tolerate a high concentration of AFB_1_, but also, their fermentation supernatant can disrupt the mycelia of *A. flavus*, which may affect the spore production and toxin synthesis of *A. flavus*.

### 2.6. The Potential of B. subtilis E11 as a Natural Food Preservative for Dried Red Chili

**Figure 6:**
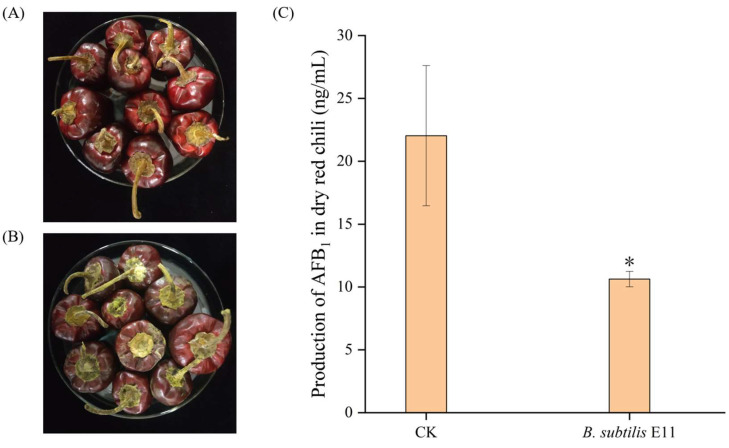
Biocontrol of *B. subtilis* E11 on dried red chili. (**A**) Dried red chili inoculated with *A. flavus* and *B. subtilis* E11; (**B**) Dried red chili inoculated with *A. flavus* without *B. subtilis* E11 as the control group; (**C**) Effect of *B. subtilis* E11 on reducing the yield of AFB_1_ in dried red chili. CK indicated the control group. Values were expressed as means ± SD; Values in the column followed by one asterisk were significantly different from those in the control group (*p* < 0.05).

The biological control ability of *B. subtilis* E11 was confirmed on dried red chili (Figure 6). After coculture for 10 d, the dried red chili in the control group was covered with *A. flavus* mycelia and spores on the carpopodium. However, only a very small number of mycelia appeared on the surface of chili treated with a culture solution of *B. subtilis* E11 (Figure 6A,B). Figure 6C illustrates the content of AFB_1_ in dried red chili after 10 days of coculture with *A. flavus* and *B. subtilis* E11 (water was used as a control). It was indicated that the yield of AFB_1_ in dried red chili could be significantly reduced after *B. subtilis* E11 treatment of chili samples. This showed that *B. subtilis* E11 has the potential to be used as a biological control agent in the storage of dried red chili. *A. flavus* is one of the common harmful fungi in dried red chili. The results of the present study may provide theoretical support for extending the storage period of dried red chili.

## 3. Discussion

According to the report of the Rapid Alert System for Food and Feed (RASFF) [43], contamination caused by AFTs accounted for about 36% of the export safety warning of dried pepper in China. Consequently, it is urgent to find a green environmental protection method to control the contamination of dried red chili by *A. flavus*. At present, scholars are committed to controlling the production of mycotoxins through biological methods, which can not only protect crops from fungal infection but also reduce damage to the ecological environment [44].

In our study, the *B. subtilis* E11 we screened was not only environmentally friendly but also, as an antagonistic bacterium, it could firstly inhibit the growth of *A. flavus* and secondly remove the AFB_1_ when *A. flavus* grew and produced it. Our results indicated that the three strains of *B. subtilis* have a stronger ability to resist *A. flavus* than some previously reported strains. For instance, a total of 10 bacterial strains were isolated from the rhizosphere soil of *Camellia sinensis* by Wu et al. [45] in the plate confrontation test, and the results showed that only *Bacillus tequilensis* and *Bacillus velezensis* had significant inhibitory effects on the growth of *A. flavus*, with inhibition rates of 34.22% and 35.72%, respectively. Another study achieved the inhibition rates of 4–90% and 5–92% for 250–2500 μL Bacillus amyloliquefaciens (UTB2) and B. subtilis (UTB3) cell culture filtrate on the growth of *Aspergillus parasiticus*, respectively [46]. Other strains, such as *Candida nivariensis* DMKU-CE18, could inhibit the growth of 64.9 ± 7.0% of *A. flavus* A39 mycelia [47]. It was revealed that the three strains of *B. subtilis* could effectively inhibit the growth of more than 60% of *A. flavus* hyphae by measuring the diameter of *A. flavus* in the control group and the experimental group.

Studies have pointed out that *Bacillus* can produce various secondary metabolites and lytic enzymes in the metabolic process, which play an important role in the antagonism of *Bacillus* [48]. The ability of *B. subtilis* E11 screened in this experiment to reduce AFB_1_ was higher than that of some reported strains. For example, Shu et al. [49] isolated a strain from soil samples named *Bacillus velezensis* DY3108, which had a strong AFB_1_ degradation activity (91.5%). In Fang et al.’s study [50], the rate of AFB_1_ degradation at 72 h was 88.59% by *Aspergillus niger* RAF106. Petchkongkaew et al. [51] found that one strain of *Bacillus licheniformis* could simultaneously inhibit the growth of *A. flavus* and *Aspergillus westerdijkiae*, and it could remove 74% of AFB_1_ and 92.5% of ochratoxin A (OTA), while another strain of *B. subtilis* could inhibit the growth of *A. flavus* and degrade AFB_1_ (85%). In another study, when 2 μg/mL AFB_1_ was added to the lysogeny broth (LB) medium, *Bacillus amylolyticus* WE2020 could reduce 70.22% of AFB_1_ at 48 h, degraded more than 84% of AFB_1_ at 72 h, and reached the maximum value [52]. A strain of *Bacillus cereus* XSWW9 was isolated from Daqu of Baijiu, which could degrade 86.7% of AFB_1_ (1 mg/L) after incubation at 37 °C for 72 h [53]. A strain of *Microbacterium proteolyticum* B204 was isolated from bovine feces, which could degrade 77% of AFB_1_ at 24 h [54]. The reported antifungal compounds produced by *B. subtilis* mainly include lipopeptides, such as surfactin, iturins, and fengycin [39,55]. *B. subtilis* is one of the most commonly used microorganisms in the industry for producing lipopeptides, which have wide applications in biology (anti-bacterial, anti-fungal, anti-viral, and anti-tumor), chemical, agricultural, pharmaceuticals, and food industries [56]. Bertuzzi et al. [48] analyzed the lipopeptides in raw and cell-free *B. subtilis* QST 713 broth by LC-MS/MS, and found that the contents of surfactants, iturins, and fengycin family in the broth were 292 mg/L, 3034 mg/L, and 1033 mg/L, respectively. However, the content of all three lipopeptides in cell-free broth was lower than that in raw broth. In addition, they also found that raw *B. subtilis* broth can completely inhibit the production of AFB_1_. As *B. subtilis* E11 exhibited to have a strong ability to degrade AFB_1_ that might be a potentially detoxification strain, the characteristics of detoxification of AFB_1_ by *B. subtilis* E11 need to be further explored. In the present study, it was only found that *B. subtilis* E11 could detoxify AFB_1_, while the antagonistic substances of *B. subtilis* E11 were not determined. Therefore, the main active substances of *B. subtilis* E11 for detoxifying AFB_1_ should be identified in the future research.

Our study found that the ability of different components of *B. subtilis* E11 to remove AFB_1_ was in the order of fermentation supernatant, cells, and cell contents. Similar results have been previously reported. For instance, the cell-free extract of *B. subtilis* fermentation could reduce AFB_1_ by 60% [28]. A strain of *Bacillus albus* YUN5 was isolated from fermented soybean paste in Korea. Through the degradation experiment of AFB_1_, it was found that the cell-free fermentation supernatant of *Bacillus albus* YUN5 had the highest degradation ability, reaching 76.28 ± 0.15%. However, the degradation ability of AFB_1_ by cells and intracellular components was negligible [57].

Next, we analyzed the expression of three key genes in the AFB_1_ synthesis pathway. Our results were similar to some published articles. Similarly, 20 strains of *Aspergillus niger* isolated from peanuts showed that the culture filtrate of *Aspergillus niger* could upregulate the expression level of the *aflR* gene while downregulating the expression levels of *aflS* and *aflD* genes [58]. Kong et al. [59] pointed out that AflR and AflS could exert a transcriptional regulatory effect on the synthetic gene cluster as a protein complex, including four AflS with one AflR. When *aflS* is expressed normally, there are sufficient AflS proteins to bind to AflR to activate the complex to regulate aflatoxin synthesis. Transcription of toxin synthetic clustered structural genes is also downregulated when the *aflS* expression level is insufficient for complex formation. In the present study, the fermentation supernatants of *B. subtilis* E11 might reduce the AFB_1_ production by upregulating the *aflR* expression level and downregulating the *aflS* expression level, thereby changing the *aflR*/*aflS* ratio. Although an *aflR*/*aflS* ratio above 1 may lead to the activation of AFB_1_ biosynthesis, it was found in some studies that AFB_1_ accumulation would not occur when the ratio was greater than 1 [60,61].

*A. flavus* is an opportunistic plant pathogen and a human pathogen [62], negatively influencing crop consumers’ health [63]. Chili may be contaminated by *A. flavus* at any stage, from field to storage. However, AFB_1_ is very difficult to remove once present in the chili production chain [2]. So, we investigated the ability of *B. subtilis* E11 as a biocontrol agent on dried red chili. The results showed that E11 not only inhibited the growth of *A. flavus* on dried chili but also reduced the content of AFB_1_. Currently, there are many successful cases of using microorganisms as biological control agents in food. For instance, it was demonstrated that *Bacillus velezensis* HC6 could simultaneously inhibit the growth of *A. flavus*, *Aspergillus ochraceus*, *Fusarium oxysporum*, *Fusarium graminearum*, *Aspergillus sulphureus*, and *Aspergillus parasiticus* in maize, and *Bacillus velezensis* HC6 could significantly reduce the contents of AFB_1_ and OTA [64]. Shakeel et al. evaluated the potential of *Streptomyces yangliansis* 3-10 as an antifungal agent on peanuts. It was found that different concentrations of culture filtrate and crude extract of *Streptomyces yanglinensis* 3-10 could inhibit the growth of *A. flavus* and reduce the production of AFB_1_. Moreover, it could significantly inhibit the expression levels of *aflR* and *aflS* during AFB_1_ synthesis. The ultrastructural changes of *A. flavus* treated with *Streptomyces yangliansis* 3-10 culture filtrate were observed by SEM and transmission electron microscopy (TEM). It was revealed that the hyphae grew abnormally and atrophied, the organelles degenerated and collapsed, and large vacuoles appeared [65]. Furthermore, 28 microorganisms isolated from red grapes to test their antifungal and anti-mycotoxigenic abilities showed that when strain UTA6 was used as a biological preservative in grapes contaminated by *A. flavus* and *Botrytis cinerea*, the spores of *A. flavus* and *Botrytis cinerea* in grapes were reduced by 0.4 and 0.6 log_10_ spores/g in grapes, while those of AFB_1_ and fumonisin B1 (FB1) decreased by 28–100% [66]. In the present study, the culture of *B. subtilis* E11 reduced the yield of AFB_1_ by 51.79%. In the subsequent study, the fermentation conditions of *B. subtilis* E11 will be optimized to enhance its ability to reduce the production of AFB_1_. On the other hand, Shifa et al.’s findings [67] will be valuable, and it will be attempted to consider applying *B. subtilis* E11 to the growth of chili in the field and actual warehouse storage. 

Overall, the *B. subtilis* E11 we have screened has great potential for application in the storage of dried red chili. Firstly, *B. subtilis* E11 is a GRAS bacterium. Secondly, *B. subtilis* E11 has a fast growth rate and low nutritional requirements, which can reduce costs in practical applications. Finally, *B. subtilis* E11 can effectively inhibit the growth of *A. flavus* and reduce the production of AFB_1_ in vitro and on dried red chili. Hence, the results of this study can provide a theoretical basis for extending the shelf life of dried red chili.

## 4. Materials and Methods

### 4.1. Fungal and Bacterial Strains

The *A. flavus* was purchased from China General Microbiology Culture Center (CGMCC; Shanghai, China), and the strain number was CGMCC 3.4408. In total, 63 bacterial strains, including 25 LAB and 35 *B. subtilis*, were isolated from fermented food and preserved in our laboratory. The other 3 LAB strains were purchased from CGMCC (CGMCC 1.2427) and China Center of Industrial Culture Collection (CICC 6239 and CICC 24,450).

### 4.2. Preparation of Spore Suspension

A spore suspension was prepared according to the method described by Natarajan et al. [68] with some modifications. *A. flavus* was transferred to the Potato Dextrose Agar (PDA) slants and incubated at 28 °C for 3–7 days. Then, sterile saline (10 mL) was utilized to wash the spores from the slants by rubbing the surface with a sterile glass rod and filtered through 4 layers of sterile gauze to obtain spore suspension. At last, the spore suspension was adjusted to 10^7^ CFU/mL using a hemocytometer under a microscope (Olympus, Tokyo, Japan) with sterile saline and used in all experiments.

### 4.3. Screening of Strains with Resistance to A. flavus

First, LAB and *B. subtilis* were cultured overnight at 37 °C on Man Rogosa and Sharpe (MRS) and LB plates, respectively. Then, 100 μL spore suspension was evenly plated on the PDA, and the single visible colony of 63 bacteria was picked up on the PDA. Each plate was equally spaced with 8 strains. The plates were incubated at 28 °C for 3–5 days. Bacteria with antifungal capacity were selected for the following experiments.

The rescreening of the antifungal ability of the strains was carried out using the method described by Zhang et al. [69] with minor variations. A 10 mm filter paper disc incubated with 30 μL of the *A. flavus* spore suspension was placed on the center of the PDA. Afterward, 30 μL of bacterial culture and sterile water aliquots were independently inoculated onto sterile filter paper discs that were placed at 4 equal distance positions, 25 mm away from the center of the PDA. The colony diameter of *A. flavus* was measured by a vernier caliper at 7 days after incubation at 28 °C. All the experiments were performed in triplicate. The inhibition rate (%) was calculated as follows: Inhibition rate (%) = (d − d’)/d × 100%, where d (mm) represents the diameter of the *A. flavus* colony in the control group, and d’ represents the *A. flavus* colony of the tested bacteria.

### 4.4. Ability of the Selected Strains to Degrade AFB_1_

The stock solution of AFB_1_ (MACKLIN, Shanghai, China) at 10 mg/L concentration was prepared in acetonitrile and kept at −20 °C. The ability of each strain to remove AFB_1_ was based on the method presented by Zhu et al. [41] with some variations. Each strain was inoculated at 37 °C for 12 h in the LB medium, followed by inoculation (5% *vol*/*vol*) of each strain into 50 mL LB medium at 37 °C for 48 h. When optical density (OD) at a wavelength of 600 nm for each strain was adjusted to make consistency, 29.55 mL inoculum (an equal volume of LB medium was used as control) was transferred to a 100 mL sterile conical flask and mixed with 0.45 mL of AFB_1_ (10 mg/L). The final concentration of AFB_1_ was 150 μg/L. Subsequently, the mixture was incubated at 28 °C with agitation at 150 rpm. The inoculums were taken at 24, 48, 72, 96 and 120 h to investigate the ability of selected strains to decrease AFB_1_ at different time points. According to the manufacturer’s instructions, a commercial enzyme-linked immunosorbent assay kit (ELISA kit, MM-092501, Jiangsu Meimian, Industrial Co., Ltd., Jiangsu, China) was utilized to determine the AFB_1_ removal capacity of the selected strains. A microplate reader (Thermo Fisher Scientific, Waltham, MA, USA) was used to measure the OD at a wavelength of 450 nm. The ability of the selected strains to degrade AFB_1_ was calculated using the following formula: (concentration of AFB_1_ in the control group-concentration of AFB_1_ in the treatment group)/concentration of AFB_1_ in the control group ×100%.

### 4.5. Determination of Growth Curves of B. subtilis E11, B. subtilis 9932, and B. subtilis V1J1

The OD 600 of 3 *B. subtilis* strains was measured using a UV-Visible spectrophotometer (UV-2100; Guangzhou, China). According to the method proposed by Ju et al. [70], *B. subtilis* E11, *B. subtilis* 9932, and *B. subtilis* V1J1 were inoculated in 5 mL liquid LB medium for activation for 2 generations, and then, the 3 *B. subtilis* strains were respectively inoculated into a 50 mL liquid LB medium (5% *vol*/*vol*) at 37 °C, 180 rpm, and OD600 was measured every 2 h.

### 4.6. AFB_1_ Biodegradation by Culture Supernatant, Cells, and Cell Lysates

The culture supernatant, cells, and cell lysates were prepared according to the method presented by Wang et al. [71] with minor modifications. First, *B. subtilis* E11 was cultured in an LB medium at 37 °C for 24 h and was then cultured (5% *vol*/*vol*) in a 50 mL LB medium at 37 °C for 48 h. The supernatant and cells were collected after centrifugation at 8000× *g* for 10 min at 4 °C, and a 0.22 µm filter was then utilized to filter the supernatant for subsequent experiments. The cell pellet was thrice washed with phosphate-buffered saline (PBS, pH = 7) and then resuspended in PBS. The cell suspension was divided into 2 parts, 1 without any treatment served as cells for AFB_1_ degradation, and the other was broken by ultrasonicator. The cell lysates were obtained after centrifugation at 10,000× *g* for 30 min at 4 °C and filtered through a 0.22 μm filter. To explore the AFB_1_ degradation, the culture supernatant, cells, and cell lysates were treated with AFB_1_ (final concentration was 150 μg/L) and were then incubated at 37 °C for 24 h with agitation at 150 rpm. The biodegradation of AFB_1_ was investigated by ELISA, according to Section 4.4.

### 4.7. The Effects of B. subtilis E11 Culture Supernant on the Expression Levels of Genes Associated with AFB_1_ Production of A. flavus

#### 4.7.1. Preparation of Mycelia Samples

Samples were prepared according to Zhu et al.’s method [41] with minor modifications. In short, 15 mL of E11 fermentation supernatant (the fermentation supernatant was collected as described in Section 4.6, and 15 mL of LB medium was used as a control) and 1.5 mL of *A. flavus* spore suspension were added to 15 mL of Potato Dextrose Broth (PDB), respectively, and the mixture was then incubated at 28 °C for 72 h with agitation at 150 rpm.

#### 4.7.2. Extraction of Total RNA

The mycelia, after 72 h of culture, were ground with liquid nitrogen. The extraction of total RNA was performed according to the instructions of the Fungal RNA kit (Omega, New York, NY, USA). The concentration and quality were subsequently measured using a NanoDrop spectrophotometer (Thermo Fisher Scientific), and the qualified samples were stored at −80 °C for subsequent experiments. 

#### 4.7.3. Synthesis of cDNA

The cDNA synthesis was performed using the StarScript II RT Mix with gDNA Remover kit (GenStar, Beijing, China). The first step was to remove residues of genomic DNA from the RNA. Then, 2 μL of RNA, 2 μL 5 × gDNA remover buffer, 1 μL gDNA remover, and 5 μL Nuclease-free water (DEPC-treated) were added to the reaction tube. The mixture was incubated at 37 °C for 5 min after being mixed and centrifuged.

Synthesis of the first strand of the cDNA was immediately carried out. Additional 4 μL of 5 × StarScript II Buffer (The Buffer was pre-mixed with Random Primer and Oligo18 (dT) Primer.), 1 μL StarScript Enzyme Mix, and 5 μL Nuclease-free water (DEPC-treated) were added to the reaction tube after incubation. The components of the reactant were evenly mixed and centrifuged. The mixture was first incubated at 42 °C for 15 min and was then heated at 85 °C for 5 min to inactivate the StarScript Enzyme Mix. The cDNA at the end of the reaction was kept at −20 °C for subsequent experiments.

#### 4.7.4. Real-Time Quantitative Polymerase Chain Reaction (RT-qPCR)

The RT-qPCR instrument equipped with Bio-Rad CFX Manager 3.1 (Bio-Rad Laboratories Inc., Hercules, CA, USA) was used to measure the expression levels of 3 genes (*aflR*, *aflS*, and *aflD*). The housekeeping gene (*β-tubulin*) was used as an endogenous control. The primers involved in this study are presented in Table 1. Each amplification reaction comprised cDNA (2 μL), SYBR Green Supermix (GenStar, Beijing, China; 10 μL), primers (forward/reverse, 10 μM; 0.4 μL), and nuclease-free water (7.2 μL). The RT-qPCR amplification conditions included initial denaturation at 94 °C for 5 min, followed by 40 amplification cycles (at 95 °C for 15 s and at 55 °C for 30 s).

### 4.8. SEM Analysis

The SEM analysis included 2 parts. One part was to observe the ultrastructural changes of *B. subtilis* E11 after coculture with AFB_1_ at 24 h, and the other part was to observe the ultrastructural changes of *A. flavus* after coculture with *A. flavus* 3.4408 and *B. subtilis* E11 supernatant for 72 h. The ultrastructural changes of *B. subtilis* E11 and *A. flavus* were observed using the SEM. This method was partially modified by referring to Zhao et al.’s research [61]. Cultures described in Section 4.6 and Section 4.7.1 were collected and centrifuged at 8000× *g* for 10 min at 4 °C. The precipitate was then washed 4 times with sterile water for 10 min. The precipitate was subsequently fixed with 2.5% glutaraldehyde overnight at 4 °C. Next, the samples were dehydrated using gradient concentrations of ethanol (10%, 30%, 50%, 70%, 95%, 100%) for 10 min. The samples were then dried in a vacuum dryer (LABCONCO Inc., New York, NY USA), which was coated with a gold ion sputter (E-1010; Hitachi, Tokyo, Japan) and examined using the SEM (S-3400N; Hitachi).

### 4.9. The Biocontrol of B. subtilis E11 on Dried Red Chili

The biocontrol of *B. subtilis* E11 on dried red chili was conducted by the method proposed by Einloft et al. [72] with some modifications. Denglongjiao was irradiated with ultraviolet radiation for 3 h to remove microorganisms on the surface of the chilis. Then, 20 g Denglongjiao was mixed with 1 mL of spore suspension and 1 mL of *B. subtilis* E11 culture suspension (sterile water of the same volume was used as control) in sterile PE self-sealing bags. After incubation at 25 °C for 10 days, the content of AFB_1_ in each sample was determined by ELISA as described in Section 4.4.

### 4.10. Statistical Analysis

All experiments were performed in triplicate. Analysis of variance (ANOVA) of SPSS 26 software (IBM, Armonk, NY, USA) was used to perform statistical analysis. The results were expressed as the mean ± SD. *p* < 0.05 was considered statistically significant.

## Figures and Tables

**Table 1:** The primers used for real-time quantitative polymerase chain reaction (RT-qPCR).

Gene	Sequence (5′–3′)	Reference
*β-tubulin*	F: TCTTCATGGTTGGCTTCGCT	[61]
	R: CTTGGGGTCGAACATCTGCT	
*aflR*	F: GGCATCTCCCGGACCGAT	
	R: TTGACAGGCAAGACCACGTTCGAT	
*aflS*	F: TGGTGCGACCATATTTACA	
	R: GGTTGGGTCACGAACTGTTT	
*aflD*	F: ATGCTCCCGTCCTACTGTTT	
	R: ATGTTGGTGATGGTGCTGAT	

## Data Availability

Not applicable.

## Supplementary Materials

The following supporting information can be downloaded at: https://www.mdpi.com/article/10.3390/toxins15050308/s1. Table S1: Preliminary screening of bacteria against *A. flavus*. Table S2: The abilities of *B. subtilis* E11, *B. subtilis* V1J1, and *B. subtilis* 9932 to degrade AFB_1_ at different time points. Figure S1: Rescreening of antagonistic bacteria against *A. flavus*. Figure S2: The effect of the fermentation supernatant of *B. subtilis* E11 on *A. flavus* mycelia.
