# Supplementary material for: Biocontrol Capabilities of Bacillus subtilis E11 against Aspergillus flavus In Vitro and for Dried Red Chili (Capsicum annuum L.)
Source: Toxins (Basel). 2023 Apr 26;15(5):308. doi: 10.3390/toxins15050308 (PMC10222026; doi:10.3390/toxins15050308)
Supplement: Supplementary file 1 [file toxins-15-00308-s001.zip › toxins-2324860-supplementary.pdf]

Table S1. Preliminary screening of bacteria against *A. flavus*

Strain	Identification	Antagonistic ability
2-1	*Lactobacillus plantarum*	-
3-2	*Lactobacillus alimentarius*	-
5-1	*Lactobacillus plantarum*	-
5-4	*Lactobacillus hilgardii*	-
7-2	*Lactobacillus pentosus*	-
7-4	*Lactobacillus alimentarius*	-
8-1	*Lactobacillus plantarum*	-
9-1	*Lactobacillus namurensis*	-
9-3	*Lactobacillus paracasei*	-
10-1	*Lactobacillus alimentarius*	-
13-4	*Lactobacillus alimentarius*	-
16-1	*Lactobacillus plantarum*	-
16-4	*Lactobacillus pentosus*	-
17-1	*Weissella hellenica*	-
19-1	*Lactobacillus pentosus*	-
19-2	*Lactobacillus alimentarius*	-
21-1	*Lactobacillus plantarum*	-
21-3	*Lactobacillus pentosus*	-
21-4	*Lactobacillus harbinensis*	-
21-5	*Pediococcus ethanolidurans*	-

24-1	*Lactobacillus plantarum*	-
25-1	*Lactobacillus alimentarius*	-
28-1	*Lactobacillus alimentarius*	-
29-1	*Lactobacillus alimentarius*	-
33-3	*Lactobacillus plantarum*	-
CICC6239	*Lactobacillus brevis*	-
CICC24450	*Lactobacillus brevis*	-
CGMCC1.2427	*Lactobacillus fructivorans*	-
4		-
E11		+
V1J		+
V2		+
J0		-
J1		-
Y1①	*Bacillus subtilis*	-
D2④		-
V1J2		+
V2②		-
VIJ1		+
2×1		+
2399		-

Fy1q	-
67	-
10081	-
y×10②	-
y×10②′	-
GQy8	-
1#	+
H6	+
B1⑥	-
Y1③	-
VIJ3	-
C1②	-
10063	+
H1	-
9932	+
B1-3	-
Z4-5	-
BJ3-2	-
KCCK	-
KCFZ	-
KCW	-

KCB	-

(Note: “+” denoted the strain had obvious bacteriostasis circle, “–” denoted the strain didn’t have obvious bacteriostasis circle)

Table S2. The abilities of *B. subtilis* E11, *B. subtilis* V1J1, and *B. subtilis* 9932 to degrade AFB_1 at different time points.

Strains	Time (h)				
	24	48	72	96	120
B. subtilis E11	81.34 ±3.25%	88.86 ±1.92%	92.85 ±0.48%	95.38 ±0.69%	96.28 ±0.62%
B. subtilis 9932	82.72 ±4.95%	86.89 ±0.57%	88.68 ±1.62%	89.31 ±0.41%	95.17 ±0.41%
B. subtilis V1J1	84.09 ±4.36%	89.03 ±1.00%	90.81 ±2.06%	94.73 ±1.00%	95.58 ±0.71%

The values were reported as average ± SD and were the mean of three independent analyses.

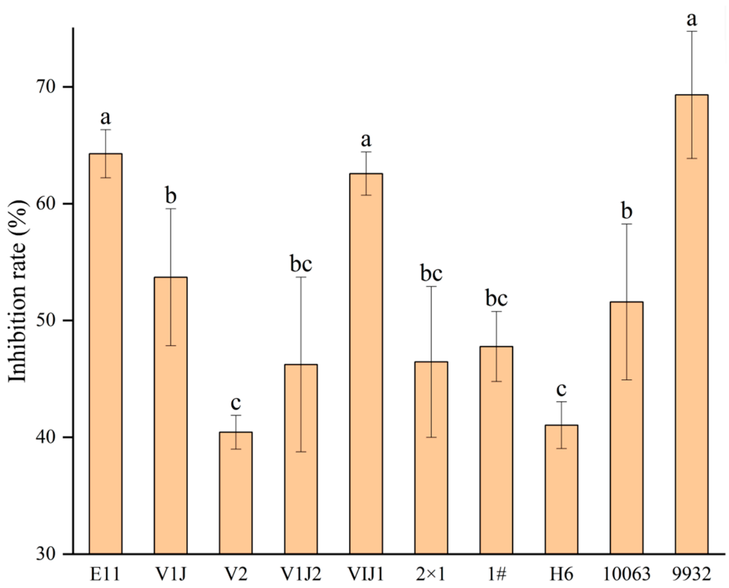

Figure S1. Rescreening of antagonistic bacteria against *A. flavus*. Values were expressed as means ± SD; a, b, c indicated significant differences of inhibition rate of different strains to *A. flavus* ($p < 0.05$).

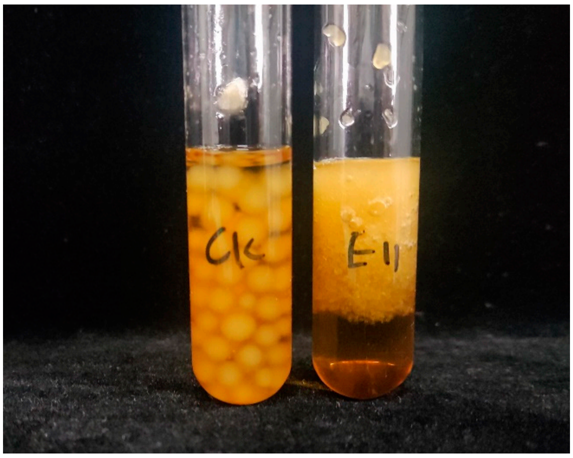

Figure S2. The effect of the fermentation supernatant of *B. subtilis* E11 on *A. flavus* mycelia. The tube on the left was the morphology of *A. flavus* mycelia after 72h culture without the fermentation supernatant of *B. subtilis* E11; The tube on the right showed the mycelia morphology of *A. flavus* co-cultured with *B. subtilis* E11 fermentation supernatant after 72h.
